# Global patterns of parasite diversity in cephalopods

**DOI:** 10.1038/s41598-020-68340-4

**Published:** 2020-07-09

**Authors:** Perla Tedesco, Stanislao Bevilacqua, Graziano Fiorito, Antonio Terlizzi

**Affiliations:** 1 Department of Veterinary Medical Sciences, Alma Mater Studiorum University of Bologna, 40064 Ozzano dell’Emilia, Italy; 2 Department of Life Sciences, University of Trieste, Trieste, Italy; 3 CoNiSMa, Piazzale Flaminio 9, 00196 Rome, Italy; 4 Department of Biology and Evolution of Marine Organisms, Stazione Zoologica Anton Dohrn, Naples, Italy; 5 Stazione Zoologica Anton Dohrn, Naples, Italy

**Keywords:** Biodiversity, Biogeography

## Abstract

We compiled an updated global catalogue of parasites in cephalopods. Data were used to assess changes in taxonomic distinctness of parasites over two centuries and across the world’s oceans, to quantify turnover and nestedness components of parasite β-diversity, and to attempt estimating their γ-diversity at a global scale. A total of 309 parasites infecting 164 cephalopods were found. We hypothesize that this diversity counts for less than half the potential parasite richness in this molluscan taxon. Taxonomic breadth of parasites was significantly above expectations from null models for Mediterranean Sea and NE Atlantic Ocean, whereas the opposite occurred for NW Pacific Ocean, where a few closely related genera characterized the parasite pool. β-diversity of parasites was very high and dominated by turnover, except for the Atlantic Ocean where a nested pattern among sub-basins emerged. Taxonomic relatedness of parasite species remained substantially unchanged through time, but species replacements largely occurred over the last two centuries. Our findings highlighted potential hotspots of taxonomic distinctness in cephalopod parasites, geographic regions deserving future research, and the need for a deeper understanding of the magnitude of marine parasite diversity, their biogeography, and their role in marine ecosystems. Our global overview may represent a baseline step for future advances in this direction.

## Introduction

With more than 800 recognized species, cephalopods inhabit a variety of marine habitats worldwide, from the intertidal zone to the deep ocean. They include species that greatly differ in terms of habitat preference, sociability (e.g., solitary vs. school-forming) and biological traits (e.g., adult body size ranging from few millimetres to meters). Several of these species are of interest to fisheries and aquaculture^1,2^ and gained special attention as model organisms in biological and medical research^3–6^. Cephalopods also play a crucial role in marine food webs^7^, both as active predators of a wide range of marine species and as prey for fish, birds and mammals.

Cephalopods harbour a variety of protozoan and metazoan parasites, which are normally acquired from the environment and through the food web. Common protozoan parasites include Ancistrocomidae ciliates, parasitizing the skin and gills of *Octopus* spp., Opalinopsidae ciliates, with members of the genus *Chromidina* parasitic in the renal appendages, and *Opalinopsis* parasitic in the liver and intestine of several Octopoda, Sepiida and Oegopsida worldwide. Coccidians of the genus *Aggregata* are also frequent, occurring with very high prevalence in the digestive tract of different species of Decapodiformes and Octopodiformes^8,9^. Members of the phylum Dicyemida, traditionally placed among the mesozoans due to their simple body plan, and recently placed among Spiralia^10^, are common but exclusively found in the renal sac of benthic cephalopods where they usually occur in heavy infections. Among metazoans, many taxa of arthropod and helminth parasites are shared between cephalopod and fish hosts, displaying low host specificity^11^, although there are examples of high specificity, such as copepods of the subfamily Cholidyinae, which includes free-living species found to infect exclusively cephalopods^12^.

Parasite diversity is often the result of multiple factors, such as habitat features, environmental changes, food preferences, migration of hosts, age and maximum size reached by the host, gregariousness or solitary behaviour^13–16^. In cephalopods, evidence suggested that the ecological niche of species is the main factor shaping its parasitic fauna^17^, although geographic factors may also play a crucial role in differentiating parasite species composition and abundance^18^. Climate change and human impacts may affect the abundance and distribution of marine parasites directly and indirectly, by influencing their free-living stages, migration patterns of their hosts^19^ or their abundance^20^. This is particularly true for heteroxenous, trophically transmitted parasites, such as anisakids, whose successful diffusion requires a wide availability of hosts throughout their life cycle and the stability of marine trophic webs^21^.

During the last decades, research in parasitology benefited from the development of new technologies and investigation techniques, which in part counterbalanced the decline in the number of expert taxonomists^22^. The increased use of molecular biology allowed resolving some uncertainties in taxonomic identifications related to the small size of parasitic organisms, and to unveil the presence of cryptic species and morphologically different life stages^23–25^. However, information on parasite diversity in cephalopods is still far from being up to date and summarized^26^.

In this study, we attempted for the first time to provide a comprehensive synthesis of parasites diversity in cephalopod hosts by compiling a list of host-parasite associations since the first documented parasitic infection in a cephalopod host, dating back to the seventeenth century^27^. Specifically, we aimed (1) to provide a picture of the magnitude, distribution, and historical trends of parasite diversity in cephalopods at a global scale. Also, we used taxonomic distinctness indices and multivariate analysis (2) to assess potential changes in taxonomic relatedness and species composition of parasites in the last two centuries, (3) to test whether the taxonomic relatedness of parasite species varied across the world’s oceans, (4) to quantify β-diversity of parasite assemblages among different biogeographic areas and periods, and (5) to explore putative host-specific associations for most widespread cephalopod genera of high ecological and commercial value. Finally, we exploited the full record to (6) attempt an estimate of γ-diversity of parasites in cephalopods worldwide.

## Results

### Taxonomic, spatial and temporal distribution of parasite records

**Figure 1:**
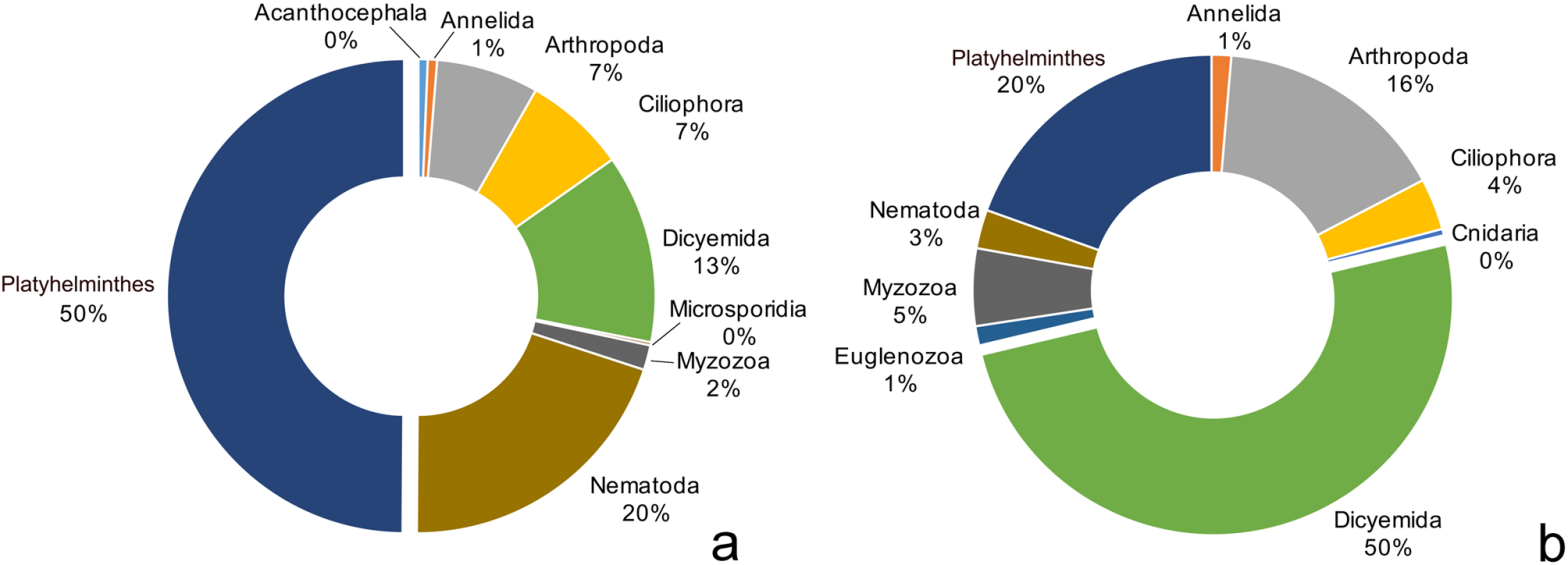
Number of host-parasite associations by parasite phyla in the two main groups of cephalopod hosts, Decapodiformes (**a**) and Octopodiformes (**b**).

A total of 492 articles were selected, reporting 695 host-parasite associations. Records span from 1684 (first record of parasitic infection in a cephalopod host) to 2017. The database includes associations involving cephalopods of the subclass Coleoidea (471 species of Decapodiformes, 224 species of Octopodiformes) and only 1 association with one species in the subclass Nautiloidea. Parasites belonged to 11 phyla, although six phyla encompassed 98% of host-parasite associations (Fig. 1). A total number of 309 parasite taxa were listed and identified in most cases at species (66%) or genus (28%) level, and occasionally at higher taxonomic levels (6%). These parasites were associated to a total of 164 cephalopod species. The number of parasite species found in a single cephalopod species ranged from 1 to 42.

The most represented parasitic phyla in terms of number of records were Platyhelminthes (275), Dicyemida (174) and Nematoda (101). Parasites infecting the highest number of cephalopod species were cestodes, with the larval form *Scolex pleuronectis* Müller, 1788 (parasitic in 38 cephalopod species), followed by *Nybelinia lingualis* Dollfus, 1929 (11), and *Tentacularia coryphaenae* Bosc, 1802 (11), and nematodes *Anisakis simplex* (Rudolphi, 1809) (22) and *Anisakis physeteris* (Baylis, 1923) (9). A higher number of parasite species was reported infecting benthic-demersal cephalopods such as Octopoda, Sepiida and Myopsida squids (263 parasite taxa) as compared to pelagic Oegopsida squids (88 parasite taxa).

**Figure 2:**
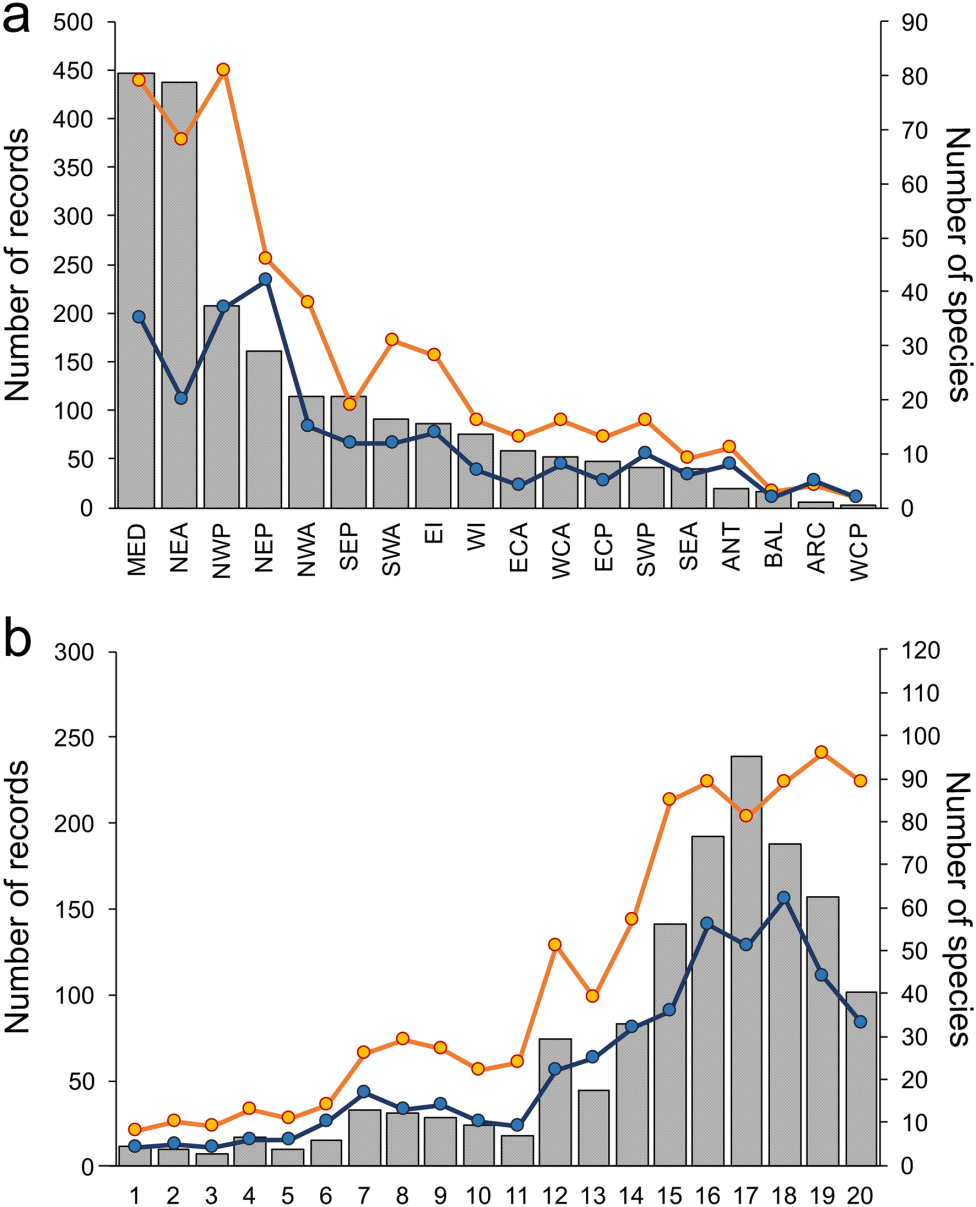
(**a**) Number of records of host-parasite associations (left *y*-axis) and number of parasite (orange line) and host (blue line) species (right *y-*axis) found in each geographic area (MED: Mediterranean Sea; NEA: NE Atlantic Ocean; NWP: NW Pacific Ocean; NEP: NE Pacific Ocean; NWA: NW Atlantic Ocean; SEP: SE Pacific Ocean; SWA: SW Atlantic Ocean; EI: Eastern Indian Ocean; WI: Western Indian Ocean; ECA: East Central Atlantic Ocean; WCA: West Central Atlantic Ocean; ECP: East Central Pacific Ocean; SWP: SW Pacific Ocean; SEA: SE Pacific Ocean; ANT: Southern Ocean; BAL: Baltic Sea; ARC: Arctic Ocean; WCP: West Central Pacific Ocean). (**b**) Number of records of host-parasite associations (left *y*-axis) and number of parasite (orange line) and host (blue line) species (right *y-*axis) found in each decade from 1817 until 2017. Numbers on the *x*-axis indicated the 20 decades.

The spatial distribution of records covers, although unevenly, almost all seas and oceans (Fig. 2a). The highest number of records was found in the Mediterranean Sea (MED), with most specimens collected in Spanish and Italian waters, followed by the Northeast Atlantic Ocean (NEA), where records come mostly from Spain and France (Fig. 2a). Records form Northwest (NWP) and Northeast Pacific (NEP) Ocean were restricted to Russia–Japan and California (USA), respectively. NWP had a number of parasite species comparable to MED and NEA, despite it accounted for about half the number of records with respect to the latter regions (Fig. 2a). Australian waters accounted for the majority of records from the Indian Ocean, while New Zealand included most of records from Southern Pacific Ocean. Studies from Argentina represented the vast majority of records in the Southwest Atlantic Ocean, while few associations were reported from the Atlantic coasts of South Africa. Records from the Central Atlantic Ocean involved almost exclusively studies carried out in Mexico. The remaining areas (Baltic Sea, Central Pacific, Southern, and Arctic Ocean) were considerably less represented.

The number of host-parasite records showed a large increase during the first half of the twentieth century and until the end of 1980s and declined in the last 3 decades, though the number of parasite species recorded remained almost unchanged (Fig. 2b). When compared with data included in the review of parasitic agents in cephalopods by Hochberg^11^, 203 new host-parasite associations were found, among which 62 included the description of new parasite species: several apicomplexans of the genus *Aggregata*, copepods (*Genesis vulcanoctopusi* Lopez-González, Bresciani and Huys in López-González, Bresciani, Huys, González, Guerra and Pascual, 2000^28^, *Amplipedicola pectinatus* Avdeev, 2010^29^ and *Doridicola similis* Ho and Kim I. H., 2001^30^), and many dicyemids. On the other hand, a considerable amount of parasite species (150) disappeared from records after 1990. These species were mostly dicyemids (40%), cestodes (16%), trematodes (12%) and copepods (11%). Missing parasites also include species, such as for instance the nematodes *Pseudoterranova decipiens* (Krabbe, 1878) Gibson, 1983 and *Hysterothylacium reliquens* (Norris and Overstreet, 1975) Deardorff and Overstreet, 1981, which disappeared from records but were found in other host organisms (e.g., fish^31,32^).

### Taxonomic distinctness of parasites from different areas and periods

**Figure 3:**
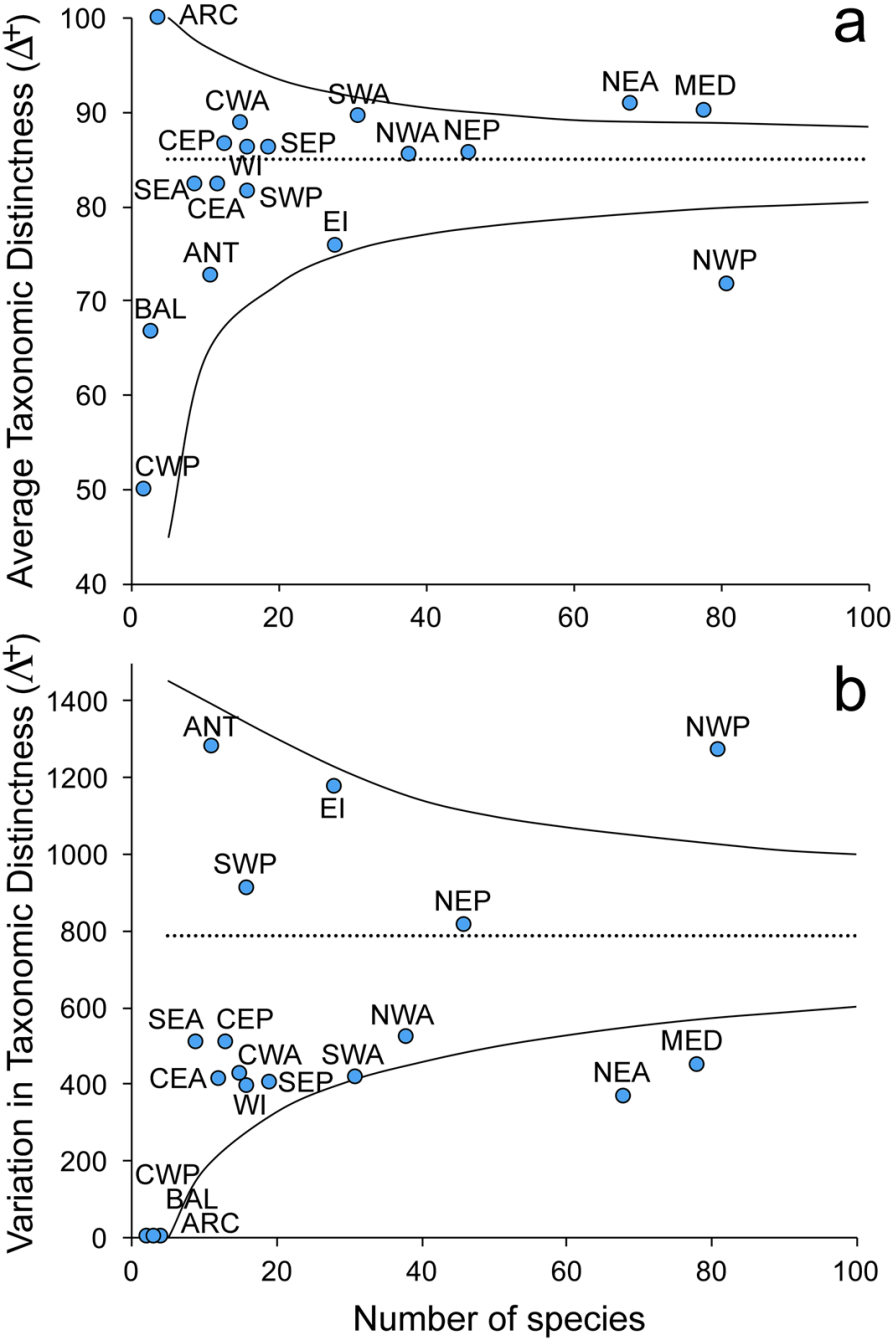
(**a**) **Δ**^**+**^ values and (**b**) **Λ**^**+**^ values of parasite assemblages recorded in each geographic area (circles), plotted against the corresponding total number of species. For both indices, the expected mean (dotted line) and the 95% confidence funnel (solid lines) were also plotted from 1,000 independent simulations of each subset (*m* = 5, 10, 20…100) drawn randomly from the list of 309 parasites species recorded (see text for details). Analysis was done using the software package PRIMER v6^70^.

The pool of parasite species characterizing different regions did not show a significant departure from random expectations of **Δ**^**+**^ and **Λ**^**+**^ values, except for MED, NEA, and NWP (Fig. 3). The former two areas had **Δ**^**+**^ values significantly higher than expectations associated to **Λ**^**+**^ values below expectations, whereas the opposite occurred in NWP (Fig. 3). Tests on **Δ**^**+**^ and **Λ**^**+**^ of cephalopod hosts from these regions suggested that the departures from expectations detected for parasite assemblages were independent from potential peculiarities of the corresponding host assemblages, which were random groups of the global pool of host species (NEA, **Δ**^**+**^ = 80.9, **Λ**^**+**^ = 414.8; MED, **Δ**^**+**^ = 79.7, **Λ**^**+**^ = 561.9; NWP, **Δ**^**+**^ = 80.3, **Λ**^**+**^ = 607.1;* P* > 0.05 in all cases).

**Figure 4:**
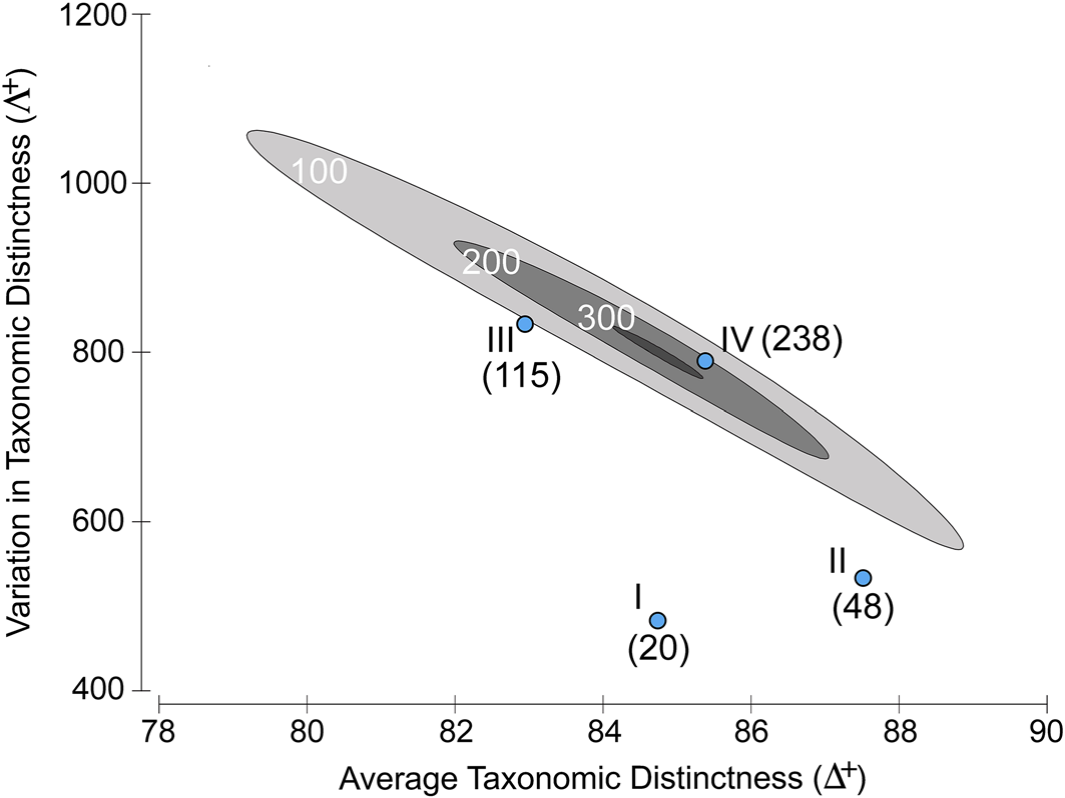
**Δ**^**+**^ values of parasite assemblages recorded in each period (I = 1817–1866, II = 1867–1916, III = 1917–1966, and IV = 1967–2016) plotted against the corresponding value of **Λ**^**+**^. Numbers in brackets indicated the total number of parasites in each period. The relevant 95% probability contours (ellipse) for (**Δ**^**+**^, **Λ**^**+**^) paired values obtained from 1,000 independent simulations from the full list of parasites were also plotted. Ellipse was built for random subsets of 300 (dark grey), 200 (grey), and 100 (light grey) species. Analysis was done using the software package PRIMER v6^70^.

Randomization tests on **Δ**^**+**^ and **Λ**^**+**^ did not detect significant changes in taxonomic structure of parasites during the last two centuries (Fig. 4). Taxonomic distinctness values of parasite assemblages in the four periods were within expectation (*P* > 0.05), indicating that the parasite species in each period were random subsets of the total pool of parasites in cephalopods.

### Global patterns of β-diversity, turnover and nestedness

β-diversity among parasite assemblages from different regions was very high, with an overall dissimilarity of 97%. The contribution of species turnover to β-diversity was dominant (94%), indicating that cephalopods from different regions were infected by completely different pools of parasites, whereas the contribution of nestedness was negligible (3%). The parasite pool from the Southeast Atlantic Ocean (SEA) was an exception to this pattern, since its dissimilarity with NEA and SWA was mostly due to nestedness (60% and 73% of the total dissimilarity, respectively), indicating that parasite species from SEA were in large part a nested subset of those found in the other sub-basins of the Atlantic Ocean (see Table S1 in Supplementary information). The lowest pair-wise dissimilarity was found comparing the East (ECA) versus West Central Atlantic Ocean (WCA), whereas the Southern Ocean (ANT) showed the highest dissimilarity values in all pair-wise comparisons (Table S1). The same patterns of β-diversity among regions characterized host assemblages (see Table S2 in Supplementary information), which showed an overall dissimilarity of 97%, dominated by species turnover (95%). β-diversity patterns between parasites and hosts assemblages from different regions were significantly correlated (r = 0.59 for overall β-diversity, r = 0.48 for turnover, r = 0.51 for the nestedness-resultant component; *P* < 0.01 in all cases), suggesting that biogeographic peculiarities in parasites species could be driven by differences in host identities among regions and species-specificity of parasite-host associations.

The overall dissimilarity in species composition of parasite assemblages among periods was high (89%) and aligned with the corresponding dissimilarity among host assemblages (86%). However, while the contribution of turnover prevailed in determining parasite dissimilarity through time (turnover component 74%, nestedness-resultant component 26%), turnover and nestedness equally participated to the overall temporal change in hosts (52% vs. 48%, respectively), indicating that the replacement of parasite species through time could not be merely related to changes in species composition of hosts (Mantel test not significant in all comparisons with *P* > 0.05, see also Table S3 in Supplementary information).

### Parasite diversity in main cephalopod genera

When analysing the taxonomic structure of parasite assemblages in the five target cephalopod genera, we found that the pool of parasites hosted by the genera *Loligo*, *Sepia*, *Illex*, and *Todarodes* fell within the random frequency distribution for both **Δ**^**+**^ and **Λ**^**+**^, except for **Λ**^**+**^ of *Loligo*, which was significantly below expectations (Table 1). In contrast, the pool of parasites of the genus *Octopus* had **Δ**^**+**^ and Λ^+^ values respectively below and above expectations (Table 1), indicating that most of recorded parasite species in *Octopus* belonged to few specific higher taxa with an uneven distribution among them. Inspection of the taxonomic list of parasites in *Octopus* revealed that ~ 60% of parasite species belonged to the class Rhombozoa (Phylum Dicyemida), and specifically to the two genera *Dicyema* and *Dicyemennea*, whereas the remaining 40% of species was distributed among 6 phyla (i.e., Platyhelminthes, Arthropoda, Nematoda, Myxozoa, Ciliophora, and Euglenozoa).

**Table 1:** Results of tests comparing **Δ**^**+**^ and **Λ**^**+**^ values calculated for parasite assemblages hosted by the cephalopod genera *Octopus*, *Sepia*, *Illex*, *Todarodes* and *Loligo*, with the corresponding frequency distribution obtained from 1,000 random simulations of a subset of the same number of species *m* from the full list of 309 parasite species recorded at global scale.

Genus	*m*	**Δ**^**+**^	*P*	**Λ**^**+**^	*P*
*Octopus*	98	74.11	**0.002**	1,227.45	**0.002**
*Sepia*	58	84.66	0.909	805.18	0.915
*Illex*	39	85.82	0.985	471.99	0.076
*Todarodes*	38	85.82	0.891	567.14	0.242
*Loligo*	26	91.85	0.060	305.31	**0.010**

Significant results are given in bold. Analysis was done using the software package PRIMER v6^70^.

The genus *Octopus* also hosted the highest number of parasite species, followed by the genus *Sepia*, whereas genera *Todarodes* and *Loligo* had lower parasite richness. Species composition of parasite assemblages greatly differed among the four investigated genera, with an overall Jaccard dissimilarity of about 95%, which was mostly due to turnover (91%) with a minor contribution of nestedness (> 4%). Only four parasite species were common to the five genera and, namely, *A. simplex*, *Phyllobothrium* sp., *Chromidina elegans* (Foettinger, 1881), and *N. lingualis*.

*Aggregata* sp., *Anisakis* sp., *Contracaecum* sp., *Pennella* sp., *Chromidina* sp., *Derogenes varicus* (Müller, 1784) Looss, 1901, *Hysterothylacium* sp., *Nybelinia* sp., and *S. pleuronectis* were shared among three genera, whereas the remaining parasite species (194) were associated to two genera or were specific of a single genus.

### A global estimate of parasite richness in cephalopods

The extrapolation of global parasite richness over 800 species of extant cephalopods, based on parasite associated to the 164 cephalopods species recorded, led to estimate a potential number of parasite species equal to 710 (± 121).

## Discussion

### Emerging geographic patterns in parasite diversity

Gaps in geographic representativeness of records make any generalization on spatial distribution of parasite diversity still premature. However, our analyses highlighted some emergent patterns, over and beyond potential bias due to the uneven occurrence of studies among regions, which could help setting next research priorities in the field. The geographical distribution of records showed areas of intense research on cephalopods parasites, such as MED, NEA and NWP, which accounted for the highest number of records and/or parasite species found. MED and NEA were also characterized by wider taxonomic range of parasites with respect to other regions. The geological history of MED and NEA probably led these regions to account for a peculiar and speciose cephalopod fauna, making them recognized hotspots and endemism-rich areas of cephalopod diversity^33^. Our findings highlighted that the high diversity of their hosts in these regions reflected into the diversification of cephalopod parasites. The high taxonomic distinctness of parasites in NEA and Med, which is not related to an analogous geographic pattern in their hosts, reinforce the evidence that these two basins can be considered at least as hotspots of parasite phylogenetic diversity. NWP, in contrast, had a significantly low value of taxonomic distinctness due to the high recurrence of species belonging to Dicyemidae. This disproportionate contribution of dicyemid to parasites diversity could depend on the activity of local research groups specialized in this taxon (i.e., Japan, Australia, for reference see^34,35^). However, the high number of dicyemid species recorded notwithstanding the relatively limited number of host species analysed, seems to indicate that this group of parasites may be more common than previously thought in cephalopods, remaining often undescribed due to the lack of specific taxonomic expertise.

Coherent biogeographic differentiation seems to underlay the overlap of β-diversity patterns between parasite and host assemblages, which were largely driven by species turnover in both cases. An exception to this general pattern was the Southeast Atlantic Ocean, where a large portion (60–70%) of the parasite pool was a subset of species from the Northeast and the Southwest Atlantic Ocean. Nestedness often originates from processes of ordered extinctions or colonizations along geographic gradients^36,37^, so that the same geographic barriers that influenced the historical distribution of cephalopods throughout the Atlantic Ocean^33^ determined the nested pattern in their parasites. However, this anomaly was independent from nestedness in host assemblages among the three regions, which was indeed negligible, indicating that diversity centres of cephalopod parasites in the north-eastern and south-western portions of the Atlantic Ocean originated from evolutionary processes in parasitic taxa, rather than to dispersal and colonization patterns of their cephalopod hosts.

### Temporal changes in the global parasite species pool

The number of parasites found in cephalopod hosts increased in time. This is not surprising since research efforts and the power to detect infections have improved due to new and more effective methodologies (e.g.,^9^). The taxonomic structure of the global parasite pool remained unchanged through time, conserving a taxonomic breadth and evenness which were undistinguishable from null expectations. More interestingly, we found that the dissimilarity in species composition of the global parasites pool over the last two centuries was almost completely driven by species turnover. As the number of studies increases and methods to detect parasites advance, one could expect that parasite species found in a given period should be, at least in part, a subset of those found in previous periods, so that nestedness should contribute substantially to the overall temporal β-diversity. This occurred, in fact, in the pool of cephalopod hosts sampled in the different periods, for which the turnover and nestedness-resultant components equally participated to the overall dissimilarity among periods. Instead, our counterintuitive findings indicated a reiterate replacement of parasite species over the last 200 years, in the absence of significant departures from expectation of taxonomic distinctness. Previous studies suggested that most of parasites species might be so rare that once they are found it could be very difficult to be encountered again in subsequent sampling^38^. The high temporal turnover in parasite assemblages and our estimate of the potential number of parasite species strongly supported this hypothesis, and clearly indicated that parasite diversity in cephalopods is far from being fully assessed.

In our view, the estimated number of about 700 parasite species could largely undervalue the actual diversity of the group by neglecting the contribute of cryptic species and micro-parasites (i.e., protozoans), which are likely to have rarely been recorded^39^.

### Parasite diversity in main cephalopod genera

Five cephalopod genera (*Illex*, *Loligo*, *Octopus*, *Sepia*, and *Todarodes*) accounted for > 50% of parasite records. A possible explanation for this high parasite diversity may simply rely on the fact that they include species representing economically valuable resources exploited by fisheries and have received high research interest^4,40,41^. However, other factors related to species behaviour and spatial distribution could have played a role in determining the exceptional parasite richness.

One potential mechanism may relate to the extent of the home range of cephalopod species. Parasites mainly rely on host movements for their dissemination^42^. Therefore, species of the genera *Illex*, *Loligo*, and *Todarodes*, which have generally large home ranges^43,44^, are likely to encounter a greater diversity of habitats and other infected individuals, which in turn may favour infection by a great number of parasite species^45^. Analogously, species with large geographical diffusion should have higher parasite diversity than those with more limited distribution. Studies carried out in terrestrial species, such as rodents and primates, have shown how their geographic range may be a good predictor of associated parasite diversity^14,46,47^. Our findings suggested that this pattern might be common also in marine organisms. This is particularly evident for cosmopolitan species of commercial interest, such as the common octopus *Octopus vulgaris* Cuvier, 1797, in which the highest number of parasite taxa was reported. Moreover, *Octopus* species have a highly diversified diet, comprising different species of crustaceans, fish and molluscs^48^ that act as intermediate hosts of parasites transmitted through trophic webs.

In spite of their high diversity in terms of species richness, parasites of the genus *Octopus* had a significantly low taxonomic breadth, indicating that the parasite species pool was more taxonomically closely related than what expected to occur by chance. This because parasites found to infect this genus, and generally all Octopodiformes, were mostly dicyemids, and often belonging to a couple of genera, namely *Dicyema* and *Dicyemennea*. These parasites are reported exclusively in the renal organs of coastal benthic cephalopods, like octopus and cuttlefish. Certain aspects of their life cycle are still enigmatic and the possible involvement of another non-cephalopod host is unknown^49,50^. In the renal sacs of cephalopods, two types of dicyemid embryos are produced: the vermiform embryo generated asexually and the infusoriform embryo that develops from a fertilized egg. The latter stage has the ability to escape from the cephalopod into the sea to search for a new host^34^. Interestingly, Dicyemida are poorly represented in Decapodiformes, which instead showed a higher proportion of metazoan parasite species. Differently from Octopodiformes, most of which are either benthic or demersal, the superorder Decapodiformes includes many pelagic species, therefore the lower proportion of dicyemids found in this group may be either related to a limited dispersal ability of infusoriform embryos in the marine environment or to the absence, in the pelagic environment, of other possible hosts involved in the dicyemid life cycle. Oceanic squids are important hosts in the life cycle of Didymozoids (*Monilicaecum* sp., *Torticaecum* sp.), Trypanorhynchs (*Nybelinia* spp., *Tentacularia coryphaenae*) and for an assemblage of poorly known species of cestodes belonging to the order Phyllobothriidea (*Phyllobothrium* spp., *Pelichnibothrium* spp.)^51^, but also of different anisakid species, especially in the genus *Anisakis*. Squids belonging to the families Onychoteuthidae and Histiotheuthidae are the preferred prey items of beaked whales, which in turn represent the definitive hosts of the recently described species *Anisakis ziphidarum* Paggi, Nascetti, Webb, Mattiucci, Cianchi and Bullini, 1988 and *Anisakis nascettii* Mattiucci, Paoletti and Webb, 2009, suggesting that the life cycle of these species might be related to cephalopods as intermediate hosts^20^.

### Final remarks

Most oceans and seas, including potential hotspots of parasite diversity, merit further investigations to achieve a more comprehensive understanding of the magnitude of marine parasites diversity. The Western Central Pacific Ocean, for instance, was the least studied region deserving priority research investments, considering that the tropical indo-pacific zone represents a global centre of cephalopod diversity^33^ and of the overall marine biodiversity^52^. Polar regions were also poorly studied, and given the high peculiarity of parasite species pool that emerged analysing the record from the Southern Ocean, a substantial contribution to parasite diversity from research in these regions can be envisaged.

A major impediment for an exhaustive quantification of parasite diversity in cephalopods, and other marine organisms, is the scant knowledge on many speciose parasitic taxa. This is particularly true for cestode larvae belonging to the orders Tetraphyllidea and Onchoproteocephalidea, which are difficult to identify at species level based on the sole morphology, such as the parasites of the genus *Scolex* and *Phyllobothrium*, but also for the *Anisakis simplex* species complex. For instance, *Scolex pleuronectis*, infecting several cephalopod species, is believed to represent a group of species belonging to different genera infecting a wide range of bony fish, cephalopods and gastropods worldwide^41,53^. Members of this taxon are further classified based on the number of loculi in the bothridia (*unilocularis, bilocularis, trilocularis,* or *quadrilocularis*^53^), contributing to increase confusion in taxonomic identifications. Future research should focus on molecular characterization of larval marine cestodes in order to increase the efforts in describing their life cycle throughout different hosts and resolve the taxonomic uncertainty about this group of marine parasites^54,55^. Problems related to taxonomic resolution also concern different protozoan parasites, particularly those belonging to the phyla Myzozoa, Ciliophora and Euglenozoa, among which the genus *Aggregata* (Myzozoa) is an emblematic example. In the full dataset we collected, only ten species of *Aggregata* were identified, each of them reported to infect a specific cephalopod species, while in the other cases these parasites were reported at the level of genus. Since marked host-specificity denotes the genus *Aggregata*, with preliminary molecular evidence also suggesting the occurrence of genetic differentiation of *Aggregata* species in different populations of their hosts^9^, detailed taxonomic analyses are needed to unveil the potential diversity hidden within this group of parasites.

For long time the ecological function of parasites has been overlooked, though their importance in maintaining biodiversity, regulating species coexistence, and modifying trophic interactions is undeniable^56–58^. In the marine realm, cephalopods are key ecological components of open sea and coastal ecosystems, with high socio-economic value for humans. Understanding the role of parasites in population dynamics, behaviour, and interspecific interactions of these molluscs is therefore essential^26^. Our overview of parasite diversity in cephalopods, by collecting and organizing parasite-host association records over the last centuries at a global scale, may represent a baseline step for future advances in this direction.

## Methods

### Data collection

Data on cephalopod-parasite associations were compiled by searching through the ISI web of Science (www.webofknowledge.com), the Natural History Museum Host-Parasite Database (www.nhm.ac.uk), and CAB Abstracts database (www.cabdirect.org). Additionally, the Biodiversity Heritage Library (www.biodiversitylibrary.org), and the Library of the Stazione Zoologica Anton Dohrn (Napoli, Italy), were searched for older literature. Reports of infection of viral, bacterial and fungal agents were not considered. Databases were queried searching the keywords ‘cephalopod(s)’ and ‘parasite(s)’ in the topic field. The list of articles was then refined by selecting relevant ones based on their title and abstract. Finally, the selected articles were scanned to extract information on the parasite and host species found, location, and year of record. The full list of host-parasite associations and literature references is provided as Supplementary information (Appendix S1). Full taxonomic details of parasites and hosts were checked through the World Register of Marine Species (www.marinespecies.org) and species names revised if necessary. In very few cases (15 species in total), it was not possible to resolve invalid species names (see Appendix S1), which were reported as provided in the related article. The potential effect of this taxonomic issue on subsequent analyses, however, can be considered as negligible, since the occurrence of these taxa did not inflate specific regions, or periods, or cephalopod taxa.

### Statistical analyses

The Average Taxonomic Distinctness^59^ and Variation in Taxonomic Distinctness^60^, and associated randomization tests, were used to assess patterns of variations in the taxonomic structure of parasite assemblages in relations to historical changes, geographic distribution, and to investigate host-specific associations between parasites in main cephalopod genera. The Average Taxonomic Distinctness (Δ^+^) represents the average taxonomic path length between two randomly chosen species in the taxonomic tree, whereas Variation in Taxonomic Distinctness (Λ^+^) reflects the unevenness of distribution of species within higher taxa and, therefore, the variance of these pair-wise path lengths. These indices have been applied to explore taxonomic diversity of several organisms in different environmental and geographic contexts around the world (e.g.,^61–63^), including the study of host-parasite associations (e.g.,^64,65^). Δ^+^ and Λ^+^ are independent from the number of species and virtually not influenced by sample size^66,67^, representing optimal tools when analysing incidence-based historical data (e.g.,^68^).

A taxonomic list, from species to phylum (including genus, family, order, subclass and class), was compiled with all parasite species of cephalopods recorded in the last two centuries from 1817 until 2017. Taxa identified at genus or coarser taxonomic level were considered as separate species. This list was used as a reference to calculate taxonomic distinctness indices and perform the associated statistical tests. All records of parasite species were organized in a data matrix including four subsets of data corresponding to 50-years periods from 1817 until 2017, so to have at least 50 records for each period. A second data matrix of parasite species (across the two centuries) was organized to include subsets of data corresponding to different geographic regions of recording and, namely, Northeast Atlantic (NEA), Northwest Atlantic (NWA), Western Central Atlantic (WCA), Eastern Central Atlantic (ECA), Southwest Atlantic (SWA), Southeast Atlantic (SEA), Western Indian (WI), Eastern Indian (EI), Northwest Pacific (NWP), Northeast Pacific (NEP), Western Central Pacific (WCP), Eastern Central Pacific (ECP), Southwest Pacific (SWP), Southeast Pacific (SEP), Southern (ANT) and Arctic (ARC) Ocean, Mediterranean (MED) and Baltic (BAL) Sea. This subdivision avoided excessive geographic fragmentation of records in the analysis, but it was still largely coherent with marine ecoregions proposed by Spalding et al.^69^.

The reference list of parasites species and the two data matrices were used to calculate Δ^+^ and Λ^+^ values for each period and in each region, which were tested to assess significant changes during the investigated time span or differences among regions in the taxonomic structure of parasite assemblages. To test the departure from expectations of indices calculated for regional subsets (under the null hypothesis that they were equally sized random subsets of parasite species from the whole reference list), the 95% ‘confidence funnel’ was obtained for Δ^+^ and Λ^+^^60^. This procedure generated 1,000 random subsets of *m* species from the reference list, calculated the index values and defined the corresponding 95% confidence interval. Randomizations were then repeated over a range of *m* values to obtain the confidence funnels against which testing the true values. Based on the same logic of funnels, a scatter plots of simulated Δ^+^ against the Λ^+^ values can be constructed, generating a combined 95% confidence ellipse for a specified number of species^60^. This bivariate approach was used to test the departure from expectations of both Δ^+^ and Λ^+^ values calculated on parasite assemblages from each historical period. For calculations of indices and 95% confidence intervals, the same step length (equal to 1) was used in weighting all distances between hierarchical taxonomic levels^70^.

Since the analysis highlighted significant departures from expectation of taxonomic distinctness of parasites for some regions (see “Results” section), an analysis of taxonomic distinctness of associated host assemblages was performed to ascertain whether differences observed in parasites could be simply a result of geographic patterns in taxonomic distinctness of their hosts. We compiled a full inventory of cephalopod hosts recorded in the last two centuries, calculated Δ^+^ and Λ^+^ of cephalopod assemblages in each region and tested their values following the same procedure described for parasites.

Patterns of β-diversity of parasite assemblages in space and time were investigated by calculating the overall Jaccard dissimilarity among regions, and among the four periods. The overall β-diversity was partitioned into the two components of turnover and nestedness^71^, to understand whether changes in species composition of parasites were mostly due to replacement or loss/gain of species. The same spatial and temporal patterns of dissimilarity were quantified also for hosts, and the Mantel test was used to assess correlations with those characterizing parasite assemblages. BAL, ARC, and CWP were excluded from this analysis due to the very low number of parasites recorded in these regions.

Due to their importance as commercial fishing targets and their relatively wide distribution, we carried out a specific analysis on five cephalopod genera, and namely *Illex*, *Loligo*, *Octopus*, *Sepia*, and *Todarodes*, which altogether accounted for > 50% of parasite records. We used randomizations tests on Δ^+^ and Λ^+^ to assess whether parasites recorded for each of these genera belonged to specific taxonomic groups of parasites. We also calculated Jaccard dissimilarities among parasite assemblages characterizing each genus and quantified the contribution of turnover and nestedness to the overall dissimilarity.

Finally, the full record of parasite-host associations was used to extrapolate parasite total richness over the number of existing cephalopods, which account approximately 800 species^72–75^. We used parasites associated to the 164 cephalopod species in the full record as a sample of parasite species richness in cephalopods. In order to estimate the expected number of parasite species that would be found in a larger sample corresponding to all extant cephalopods, we employed a non-parametric extrapolation method^76^. This procedure is based on the Bernoulli product model and allows extrapolation for sample-based incidence data, providing reliable upper and lower bound 95% confidence interval, particularly when sample size on which species richness is extrapolated is not very large with respect the size of the reference sample^76^, as in our case.

All analyses were done using the software package PRIMER v6^77^, R^78^ using the package ‘betapart’^79^, and the computer program EstimateS^80^.

## Data availability

All data generated or analysed during this study are included in this published article (and its Supplementary Information files).

## Supplementary information

Supplementary Information 1.

Supplementary Information 2.
