# Supplementary material for: Global patterns of parasite diversity in cephalopods
Source: Sci Rep. 2020 Jul 9;10:11303. doi: 10.1038/s41598-020-68340-4 (PMC7347566; doi:10.1038/s41598-020-68340-4)
Supplement: Supplementary file 2 — Supplementary Information 2. [file 41598_2020_68340_MOESM2_ESM.doc]

**Global patterns of parasite diversity in cephalopods**

Perla Tedesco^1^, Stanislao Bevilacqua^2,3,*^, Graziano Fiorito^4^, Antonio Terlizzi^2,3,4^

*^1^Department of Veterinary Medical Sciences, Alma Mater Studiorum University of Bologna, Ozzano Emilia (BO) Italy*

*^2^ Department of Life Sciences, University of Trieste, Trieste, Italy*

*^3^CoNiSMa, Piazzale Flaminio 9, 00196 Roma, Italy*

*^4^Stazione Zoologica Anton Dohrn, Naples, Italy*

**Corresponding author: sbevilacqua@units.it*

**Supplementary information**

Table S1.

Table S2.

Table S3

**Table S1**. Pairwise values of compositional Jaccard dissimilarity and its turnover and nestedness-resultant components between parasite assemblages from different geographic areas. Northeast Atlantic (NEA), Northwest Atlantic (NWA), Western Central Atlantic (WCA), Eastern Central Atlantic (ECA), Southwest Atlantic (SWA), Southeast Atlantic (SEA), Western Indian (WI), Eastern Indian (EI), Northwest Pacific (NWP), Northeast Pacific (NEP), Eastern Central Pacific (ECP), Southwest Pacific (SWP), Southeast Pacific (SEP), and Southern (ANT) Ocean, Mediterranean Sea (MED). The Baltic Sea, Arctic and Western Central Pacific Ocean were not considered because of the very low number of parasites recorded in these basins. Analysis as done R [R Development Core Team. R: A language and environment for statistical computing. R Foundation for Statistical Computing, Vienna, Austria. http://www.R-project.org/ (2018).] using the package ‘betapart’ [Baselga, A. & Orme, C. D. L. betapart: An R package for the study of beta diversity. Methods Ecol. Evol. 3, 808-812 (2012).

| Overall dissimilarity | | | | | | | | | | | | | | | |
| --- | --- | --- | --- | --- | --- | --- | --- | --- | --- | --- | --- | --- | --- | --- | --- |
|  | NEA | NWA | WCA | ECA | SWA | SEA | WI | EI | NWP | NEP | ECP | SWP | SEP | ANT | MED |
| NEA |  |  |  |  |  |  |  |  |  |  |  |  |  |  |  |
| NWA | 0.84 |  |  |  |  |  |  |  |  |  |  |  |  |  |  |
| WCA | 0.91 | 0.88 |  |  |  |  |  |  |  |  |  |  |  |  |  |
| ECA | 0.91 | 0.84 | 0.47 |  |  |  |  |  |  |  |  |  |  |  |  |
| SWA | 0.86 | 0.89 | 0.85 | 0.81 |  |  |  |  |  |  |  |  |  |  |  |
| SEA | 0.9 | 0.85 | 0.81 | 0.71 | 0.75 |  |  |  |  |  |  |  |  |  |  |
| WI | 0.89 | 0.85 | 0.81 | 0.74 | 0.76 | 0.68 |  |  |  |  |  |  |  |  |  |
| EI | 0.91 | 0.88 | 0.87 | 0.83 | 0.82 | 0.81 | 0.63 |  |  |  |  |  |  |  |  |
| NWP | 0.93 | 0.95 | 0.96 | 0.96 | 0.93 | 0.95 | 0.96 | 0.96 |  |  |  |  |  |  |  |
| NEP | 0.9 | 0.89 | 0.97 | 0.96 | 0.93 | 0.94 | 0.89 | 0.91 | 0.89 |  |  |  |  |  |  |
| CEP | 0.89 | 0.81 | 0.84 | 0.76 | 0.78 | 0.53 | 0.62 | 0.76 | 0.94 | 0.89 |  |  |  |  |  |
| SWP | 0.91 | 0.88 | 0.93 | 0.93 | 0.91 | 0.86 | 0.86 | 0.9 | 0.93 | 0.97 | 0.84 |  |  |  |  |
| SEP | 0.87 | 0.79 | 0.83 | 0.77 | 0.84 | 0.73 | 0.75 | 0.83 | 0.95 | 0.93 | 0.72 | 0.75 |  |  |  |
| ANT | 0.99 | 1 | 1 | 1 | 0.98 | 1 | 1 | 1 | 1 | 1 | 1 | 1 | 1 |  |  |
| MED | 0.71 | 0.92 | 0.92 | 0.93 | 0.9 | 0.93 | 0.93 | 0.94 | 0.93 | 0.95 | 0.92 | 0.96 | 0.93 | 0.99 |  |
|  |  |  |  |  |  |  |  |  |  |  |  |  |  |  |  |
| Turnover component | | | | | | | | | | | | | | | |
|  | NEA | NWA | WCA | ECA | SWA | SEA | WI | EI | NWP | NEP | ECP | SWP | SEP | ANT | MED |
| NEA |  |  |  |  |  |  |  |  |  |  |  |  |  |  |  |
| NWA | 0.75 |  |  |  |  |  |  |  |  |  |  |  |  |  |  |
| WCA | 0.72 | 0.77 |  |  |  |  |  |  |  |  |  |  |  |  |  |
| ECA | 0.63 | 0.63 | 0.38 |  |  |  |  |  |  |  |  |  |  |  |  |
| SWA | 0.76 | 0.87 | 0.77 | 0.63 |  |  |  |  |  |  |  |  |  |  |  |
| SEA | 0.36 | 0.5 | 0.71 | 0.62 | 0.2 |  |  |  |  |  |  |  |  |  |  |
| WI | 0.67 | 0.72 | 0.81 | 0.7 | 0.61 | 0.5 |  |  |  |  |  |  |  |  |  |
| EI | 0.83 | 0.86 | 0.81 | 0.7 | 0.81 | 0.5 | 0.4 |  |  |  |  |  |  |  |  |
| NWP | 0.92 | 0.91 | 0.86 | 0.82 | 0.87 | 0.71 | 0.86 | 0.92 |  |  |  |  |  |  |  |
| NEP | 0.88 | 0.88 | 0.93 | 0.92 | 0.91 | 0.8 | 0.77 | 0.88 | 0.84 |  |  |  |  |  |  |
| CEP | 0.56 | 0.56 | 0.82 | 0.76 | 0.56 | 0.36 | 0.56 | 0.56 | 0.76 | 0.7 |  |  |  |  |  |
| SWP | 0.72 | 0.77 | 0.93 | 0.92 | 0.86 | 0.8 | 0.86 | 0.86 | 0.77 | 0.93 | 0.82 |  |  |  |  |
| SEP | 0.64 | 0.64 | 0.81 | 0.7 | 0.77 | 0.5 | 0.72 | 0.77 | 0.85 | 0.88 | 0.63 | 0.72 |  |  |  |
| ANT | 0.95 | 1 | 1 | 1 | 0.95 | 1 | 1 | 1 | 1 | 1 | 1 | 1 | 1 |  |  |
| MED | 0.68 | 0.87 | 0.72 | 0.7 | 0.81 | 0.5 | 0.77 | 0.88 | 0.93 | 0.93 | 0.63 | 0.86 | 0.81 | 0.95 |  |
|  | | | | | | | | | | | | | | | |
| Nestedness-resultant component | | | | | | | | | | | | | | | |
|  | NEA | NWA | WCA | ECA | SWA | SEA | WI | EI | NWP | NEP | ECP | SWP | SEP | ANT | MED |
| NEA |  |  |  |  |  |  |  |  |  |  |  |  |  |  |  |
| NWA | 0.09 |  |  |  |  |  |  |  |  |  |  |  |  |  |  |
| WCA | 0.19 | 0.11 |  |  |  |  |  |  |  |  |  |  |  |  |  |
| ECA | 0.28 | 0.21 | 0.09 |  |  |  |  |  |  |  |  |  |  |  |  |
| SWA | 0.1 | 0.02 | 0.08 | 0.18 |  |  |  |  |  |  |  |  |  |  |  |
| SEA | 0.54 | 0.35 | 0.1 | 0.09 | 0.55 |  |  |  |  |  |  |  |  |  |  |
| WI | 0.22 | 0.13 | 0 | 0.04 | 0.15 | 0.18 |  |  |  |  |  |  |  |  |  |
| EI | 0.08 | 0.02 | 0.06 | 0.13 | 0.01 | 0.31 | 0.23 |  |  |  |  |  |  |  |  |
| NWP | 0.01 | 0.04 | 0.1 | 0.14 | 0.06 | 0.24 | 0.1 | 0.04 |  |  |  |  |  |  |  |
| NEP | 0.02 | 0.01 | 0.04 | 0.04 | 0.02 | 0.14 | 0.12 | 0.03 | 0.05 |  |  |  |  |  |  |
| CEP | 0.33 | 0.25 | 0.02 | 0 | 0.22 | 0.17 | 0.06 | 0.2 | 0.18 | 0.19 |  |  |  |  |  |
| SWP | 0.19 | 0.11 | 0 | 0.01 | 0.05 | 0.06 | 0 | 0.04 | 0.16 | 0.04 | 0.02 |  |  |  |  |
| SEP | 0.23 | 0.15 | 0.02 | 0.07 | 0.07 | 0.23 | 0.03 | 0.06 | 0.1 | 0.05 | 0.09 | 0.03 |  |  |  |
| ANT | 0.04 | 0 | 0 | 0 | 0.03 | 0 | 0 | 0 | 0 | 0 | 0 | 0 | 0 |  |  |
| MED | 0.03 | 0.05 | 0.2 | 0.23 | 0.09 | 0.43 | 0.16 | 0.06 | 0 | 0.02 | 0.29 | 0.1 | 0.12 | 0.04 |  |

**Table S2**. Pairwise values of compositional Jaccard dissimilarity and its turnover and nestedness-resultant components between cephalopod hosts assemblages from different geographic areas. Acronyms are as in Table S1. Analysis as done R [R Development Core Team. R: A language and environment for statistical computing. R Foundation for Statistical Computing, Vienna, Austria. http://www.R-project.org/ (2018).] using the package ‘betapart’ [Baselga, A. & Orme, C. D. L. betapart: An R package for the study of beta diversity. Methods Ecol. Evol. 3, 808-812 (2012).

| Overall dissimilarity | | | | | | | | | | | | | | | |
| --- | --- | --- | --- | --- | --- | --- | --- | --- | --- | --- | --- | --- | --- | --- | --- |
|  | NEA | NWA | WCA | ECA | SWA | SEA | WI | EI | NWP | NEP | ECP | SWP | SEP | ANT | MED |
| NEA |  |  |  |  |  |  |  |  |  |  |  |  |  |  |  |
| NWA | 0.87 |  |  |  |  |  |  |  |  |  |  |  |  |  |  |
| WCA | 0.83 | 0.79 |  |  |  |  |  |  |  |  |  |  |  |  |  |
| ECA | 0.91 | 0.94 | 0.67 |  |  |  |  |  |  |  |  |  |  |  |  |
| SWA | 0.97 | 0.96 | 0.95 | 0.93 |  |  |  |  |  |  |  |  |  |  |  |
| SEA | 0.87 | 0.95 | 0.92 | 0.89 | 0.71 |  |  |  |  |  |  |  |  |  |  |
| WI | 0.92 | 0.9 | 0.85 | 0.9 | 0.94 | 0.92 |  |  |  |  |  |  |  |  |  |
| EI | 0.97 | 0.96 | 0.95 | 0.94 | 0.92 | 0.95 | 0.69 |  |  |  |  |  |  |  |  |
| NWP | 0.96 | 0.96 | 0.95 | 0.97 | 0.96 | 0.98 | 0.93 | 0.91 |  |  |  |  |  |  |  |
| NEP | 0.98 | 0.96 | 0.98 | 0.98 | 0.98 | 0.98 | 0.96 | 0.94 | 0.89 |  |  |  |  |  |  |
| CEP | 1 | 1 | 1 | 1 | 1 | 1 | 0.91 | 0.94 | 0.98 | 0.91 |  |  |  |  |  |
| SWP | 0.93 | 0.91 | 0.88 | 0.92 | 0.9 | 0.86 | 0.87 | 0.91 | 0.95 | 0.96 | 0.93 |  |  |  |  |
| SEP | 0.93 | 0.96 | 0.95 | 0.93 | 0.86 | 0.8 | 0.81 | 0.92 | 0.96 | 0.94 | 0.87 | 0.71 |  |  |  |
| ANT | 1 | 1 | 1 | 1 | 1 | 1 | 1 | 1 | 1 | 1 | 1 | 1 | 1 |  |  |
| MED | 0.69 | 0.94 | 0.93 | 0.97 | 0.96 | 0.92 | 0.95 | 0.96 | 0.96 | 0.97 | 1 | 0.95 | 0.96 | 1 |  |
|  |  |  |  |  |  |  |  |  |  |  |  |  |  |  |  |
| Turnover component | | | | | | | | | | | | | | | |
|  | NEA | NWA | WCA | ECA | SWA | SEA | WI | EI | NWP | NEP | ECP | SWP | SEP | ANT | MED |
| NEA |  |  |  |  |  |  |  |  |  |  |  |  |  |  |  |
| NWA | 0.85 |  |  |  |  |  |  |  |  |  |  |  |  |  |  |
| WCA | 0.67 | 0.67 |  |  |  |  |  |  |  |  |  |  |  |  |  |
| ECA | 0.67 | 0.86 | 0.4 |  |  |  |  |  |  |  |  |  |  |  |  |
| SWA | 0.96 | 0.96 | 0.93 | 0.86 |  |  |  |  |  |  |  |  |  |  |  |
| SEA | 0.67 | 0.91 | 0.91 | 0.86 | 0.5 |  |  |  |  |  |  |  |  |  |  |
| WI | 0.83 | 0.83 | 0.83 | 0.86 | 0.92 | 0.91 |  |  |  |  |  |  |  |  |  |
| EI | 0.96 | 0.96 | 0.93 | 0.86 | 0.91 | 0.91 | 0.44 |  |  |  |  |  |  |  |  |
| NWP | 0.95 | 0.93 | 0.86 | 0.86 | 0.91 | 0.91 | 0.73 | 0.83 |  |  |  |  |  |  |  |
| NEP | 0.97 | 0.93 | 0.93 | 0.86 | 0.96 | 0.91 | 0.83 | 0.88 | 0.88 |  |  |  |  |  |  |
| CEP | 1 | 1 | 1 | 1 | 1 | 1 | 0.89 | 0.89 | 0.89 | 0.33 |  |  |  |  |  |
| SWP | 0.89 | 0.89 | 0.86 | 0.86 | 0.89 | 0.8 | 0.83 | 0.89 | 0.89 | 0.89 | 0.89 |  |  |  |  |
| SEP | 0.91 | 0.96 | 0.93 | 0.86 | 0.86 | 0.67 | 0.73 | 0.91 | 0.91 | 0.86 | 0.75 | 0.67 |  |  |  |
| ANT | 1 | 1 | 1 | 1 | 1 | 1 | 1 | 1 | 1 | 1 | 1 | 1 | 1 |  |  |
| MED | 0.52 | 0.89 | 0.77 | 0.86 | 0.91 | 0.67 | 0.83 | 0.92 | 0.96 | 0.97 | 1 | 0.89 | 0.91 | 1 |  |
|  |  |  |  |  |  |  |  |  |  |  |  |  |  |  |  |
|  |  |  |  |  |  |  |  |  |  |  |  |  |  |  |  |
|  |  |  |  |  |  |  |  |  |  |  |  |  |  |  |  |
|  |  |  |  |  |  |  |  |  |  |  |  |  |  |  |  |
| Nestedness-resultant component | | | | | | | | | | | | | | | |
|  | NEA | NWA | WCA | ECA | SWA | SEA | WI | EI | NWP | NEP | ECP | SWP | SEP | ANT | MED |
| NEA |  |  |  |  |  |  |  |  |  |  |  |  |  |  |  |
| NWA | 0.02 |  |  |  |  |  |  |  |  |  |  |  |  |  |  |
| WCA | 0.16 | 0.12 |  |  |  |  |  |  |  |  |  |  |  |  |  |
| ECA | 0.24 | 0.08 | 0.27 |  |  |  |  |  |  |  |  |  |  |  |  |
| SWA | 0.01 | 0 | 0.02 | 0.07 |  |  |  |  |  |  |  |  |  |  |  |
| SEA | 0.2 | 0.04 | 0.01 | 0.03 | 0.21 |  |  |  |  |  |  |  |  |  |  |
| WI | 0.09 | 0.07 | 0.02 | 0.04 | 0.02 | 0.01 |  |  |  |  |  |  |  |  |  |
| EI | 0.01 | 0 | 0.02 | 0.08 | 0.01 | 0.04 | 0.25 |  |  |  |  |  |  |  |  |
| NWP | 0.01 | 0.03 | 0.09 | 0.11 | 0.05 | 0.07 | 0.2 | 0.08 |  |  |  |  |  |  |  |
| NEP | 0.01 | 0.03 | 0.05 | 0.12 | 0.02 | 0.07 | 0.13 | 0.06 | 0.01 |  |  |  |  |  |  |
| CEP | 0 | 0 | 0 | 0 | 0 | 0 | 0.02 | 0.05 | 0.09 | 0.58 |  |  |  |  |  |
| SWP | 0.04 | 0.02 | 0.02 | 0.06 | 0.01 | 0.06 | 0.04 | 0.02 | 0.06 | 0.07 | 0.04 |  |  |  |  |
| SEP | 0.02 | 0 | 0.02 | 0.07 | 0 | 0.13 | 0.08 | 0.01 | 0.05 | 0.08 | 0.12 | 0.04 |  |  |  |
| ANT | 0 | 0 | 0 | 0 | 0 | 0 | 0 | 0 | 0 | 0 | 0 | 0 | 0 |  |  |
| MED | 0.17 | 0.05 | 0.16 | 0.11 | 0.05 | 0.25 | 0.12 | 0.04 | 0 | 0 | 0 | 0.06 | 0.05 | 0 |  |

**Table S3**. Pairwise values of compositional Jaccard dissimilarity and its turnover and nestedness-resultant components between parasite assemblages from different periods. (I = 1817-1866, II = 1867-1916, III = 1917-1966, and IV = 1967-2017). Analysis as done R [R Development Core Team. R: A language and environment for statistical computing. R Foundation for Statistical Computing, Vienna, Austria. http://www.R-project.org/ (2018).] using the package ‘betapart’ [Baselga, A. & Orme, C. D. L. betapart: An R package for the study of beta diversity. Methods Ecol. Evol. 3, 808-812 (2012).

| Overall dissimilarity |  | I | II | III | IV |
| --- | --- | --- | --- | --- | --- |
|  | I |  |  |  |  |
|  | II | 0.83 |  |  |  |
|  | III | 0.83 | 0.74 |  |  |
|  | IV | 0.97 | 0.90 | 0.80 |  |
|  |  |  |  |  |  |
| Turnover component | I |  |  |  |  |
|  | II | 0.67 |  |  |  |
|  | III | 0.00 | 0.45 |  |  |
|  | IV | 0.79 | 0.65 | 0.65 |  |
|  |  |  |  |  |  |
| Nestedness-resultant component | I |  |  |  |  |
|  | II | 0.16 |  |  |  |
|  | III | 0.83 | 0.28 |  |  |
|  | IV | 0.18 | 0.26 | 0.15 |  |
